# Anxiety-Free Public Dentistry for Adults With Disabilities by Using Head-Mounted Virtual Reality Technology: Protocol for a Feasibility Mixed Methods Study

**DOI:** 10.2196/85916

**Published:** 2026-02-13

**Authors:** Dung T Bui, Fiona McAlinden, Alice Urban, Charmine E J Hartel, Kristian Rotaru, Kadek Ananta Satriadi, Tanner Person, Wei Wang, Libby Callaway

**Affiliations:** 1 School of Primary and Allied Health Care Faculty of Medicine, Nursing and Health Sciences Monash University Frankston, VIC Australia; 2 National Centre for Healthy Ageing Faculty of Medicine, Nursing and Health Sciences Monash University Frankston, VIC Australia; 3 Integrated Care and Dental Peninsula Health Frankston, VIC Australia; 4 Faculty of Business and Economics Monash University Caulfield, VIC Australia; 5 Opportunity Tech Lab Monash University Clayton, VIC Australia; 6 School of Psychological Sciences Monash University Clayton, VIC Australia; 7 Faculty of Information Technology Monash University Clayton, VIC Australia; 8 Rehabilitation, Ageing and Independent Living Research Centre Faculty of Medicine, Nursing and Health Sciences Monash University Frankston, VIC Australia; 9 Occupational Therapy Department Faculty of Medicine, Nursing and Health Sciences Monash University Frankston, VIC Australia

**Keywords:** virtual reality, dentistry, disability, inclusive health care, dental anxiety reduction, feasibility, oral health, innovation

## Abstract

**Background:**

Oral disease remains a global public health concern, disproportionately affecting socioeconomically disadvantaged populations. Adults with disabilities or health conditions face additional barriers to dental care, including physical accessibility, communication challenges, and heightened anxiety. These factors contribute to care avoidance and poorer oral health outcomes. While virtual reality (VR) has shown promise in reducing procedural anxiety in pediatric and private dental settings, its application in adult public dentistry, particularly for people with disabilities, remains underexplored.

**Objective:**

This study aims to evaluate the feasibility, usability, and acceptability of Smileyscope, a Therapeutic Goods Administration–approved head-mounted VR headset, in reducing dental anxiety and enhancing care experiences for adults with disabilities in public dental clinics.

**Methods:**

A mixed methods convergent design will be implemented across community dental sites in Victoria, Australia. In total, 50 adult patients and up to 10 dental staff members will be recruited. Primary feasibility indicators include recruitment rate (≥60% consent), completion rate (≥80% System Usability Scale [SUS] completion), and usability threshold (mean SUS ≥68). The primary analysis will be descriptive, with 95% CIs reported. Quantitative data will be collected using the visual analog scale for willingness, the SUS, and the technology acceptance model questionnaire. Qualitative data from semistructured interviews will be thematically analyzed. The study is co-designed with a disability advocate and integrates lived experience throughout all phases, including recruitment, evaluation, and dissemination.

**Results:**

The project was funded in 2025, and ethics approval was granted by Peninsula Health Human Research Ethics Committee (project ID 117565). Data collection is scheduled for October 2025 to March 2026 at the participating community dental sites. Primary feasibility estimates and qualitative themes are expected to be submitted for publication in mid‑2026.

**Conclusions:**

This protocol outlines a feasibility study that will inform scalable models for VR integration into public dental services. The findings will contribute to improved oral health equity and patient-centered care, advancing the evidence base for inclusive digital health innovation in dentistry.

**International Registered Report Identifier (IRRID):**

PRR1-10.2196/85916

## Introduction

### Background

In 2021, the World Health Assembly approved a resolution on oral care, recognizing it as a key preventive primary health care measure and setting targets for a global strategy and action plan for oral health [1]. Oral disease remains a significant global issue and disproportionately affects poor and socially disadvantaged populations. Unlike private or pediatric settings, public dental clinics face throughput constraints, variable sensory needs, and fewer standardized accommodations. These limitations can amplify anxiety and care avoidance among adults with disabilities.

Adults with disabilities, including those with different health conditions or varying levels of impairment or activity limitations [2,3], can face persistent and multifaceted challenges in accessing public dental care, including physical accessibility barriers, communication difficulties, and heightened sensory sensitivities. These challenges are compounded by systemic issues such as clinician reluctance, inadequate training in disability-inclusive care, and limited infrastructure to support tailored interventions [4,5]. Public dental services often operate under resource constraints, which can exacerbate anxiety and reduce treatment adherence among this population [6]. Compared to pediatric dental settings, in which virtual reality (VR) has shown promise in reducing procedural anxiety, adult public dentistry presents a more complex context. This includes greater variability in cognitive and physical abilities, fewer accommodations for sensory needs, and a lack of standardized protocols for VR integration [7]. Moreover, adults with disabilities or health conditions can be more likely to delay or avoid dental care due to fear, discomfort, or previous negative experiences, leading to poorer oral health outcomes [7-10]. These factors underscore a critical evidence gap: although VR has demonstrated efficacy in reducing procedural anxiety in pediatric and private dental settings, its feasibility and acceptability among adults with disabilities in resource-constrained public clinics remain largely unexplored.

As technology advances, the use of head-mounted displays for VR application–based purposes—including in the delivery of primary health care interventions such as dental care—is increasing and can offer novel approaches to patient engagement and treatment uptake [11]. The existing literature on VR technology within dental care highlights several critical points from both clinician and patient perspectives. Clinicians perceive VR as valuable for treatment planning and enhancing patient communication. Research also indicates that VR technology can facilitate the simulation of treatment outcomes and can assist in procedures such as dental implants [12,13]. Furthermore, clinicians report that VR technology can significantly enhance the patient experience by alleviating anxiety, particularly among pediatric populations and populations with disabilities. Studies indicate that VR positively affects patient anxiety levels and procedural efficiency, thereby rendering care procedures less daunting [14-17]. Clinicians regard VR as an essential tool for improving dental education and training as it can also offer immersive learning opportunities and allows for the practice of complex procedures within a controlled environment [13,18-21].

Research has demonstrated that patients similarly appreciate the contribution of VR in reducing dental anxiety and pain. This technology has previously been found to offer a distraction during procedures, thereby enhancing overall comfort [14,15,17]. Research also demonstrates notable reductions in heart rate and stress levels when VR is used as a distraction during dental interventions. Moreover, patients find value in VR’s educational offering, which aids them in comprehending treatment plans and procedures more effectively [13,22].

Despite the favorable perceptions held by many clinicians and patients regarding VR, issues have also been found. A systematic review of factors associated with VR motion sickness in head-mounted displays found that sickness profiles were influenced by both the type and duration of content used (with visual stimulation or locomotion content and increased exposure times linked to motion sickness) and the age and ability of the user [11]. From a dental clinician perspective, research trials have indicated that headset bulkiness may impact the treatments that can be administered [23].

Several significant gaps also remain in the research regarding both the perspectives of dental staff and patients on the potential of this technology in dental care. Most studies that do exist primarily focus on immediate outcomes, such as anxiety and pain alleviation during a single dental visit. There is a lack of research examining the efficacy of VR across various dental procedures and its long-term benefits, particularly regarding its impact on patient outcomes and clinician performance over extended periods [14,24]. In addition, current literature often fails to account for diversity in patient demographics, including age, socioeconomic status, and cultural background [17]. Many studies have concentrated predominantly on pediatric patients, with limited research explicitly addressing the experiences and outcomes of adult patients. This narrow focus constrains the generalizability of findings and the capacity to tailor VR interventions to diverse patient groups.

There is a pressing need for comprehensive studies addressing the level of training that clinicians receive in using VR technology and their overall receptiveness to its integration into routine dental practice [25]. Moreover, the establishment of standardized protocols and guidelines for the integration of VR in dental practices has to ensure consistency and efficacy [26]. Addressing these gaps has the potential to offer a more comprehensive understanding of how VR technology can be effectively integrated into dental care, benefitting both clinicians and adult patients.

Smileyscope (Smileyscope Pty Ltd) is a Therapeutic Goods Administration–approved VR headset (Australian Register of Therapeutic Goods ID297181) [27] designed for health care settings with compatibility with standard infection control protocols and a library of immersive VR experiences tailored to health care settings. The headset is a low-cost, easy-to-deploy head-mounted VR display that has demonstrated efficacy in reducing procedural pain and anxiety in pediatric hospital environments [28-30]. It is widely used across Australia, the United States, and the United Kingdom, with multiple independent studies validating its effectiveness in health care delivery [31,32]. While Smileyscope has been trialed in various medical contexts, including palliative care and heart failure management [33-35], its application in dental care—particularly for adults with disabilities—remains unexplored.

### Research Aim

This study aims to address the research gaps by evaluating the feasibility, acceptability, and usability of Smileyscope in public dental settings for adults, including people with disabilities (hereafter referred to as “patients”). By integrating lived experience and inclusive design principles, this study seeks to understand both patient and clinician perspectives on VR use during routine dental procedures. The findings will inform scalable models for VR integration into public oral health services, contributing to improved equity and patient-centered care, and prespecify feasibility benchmarks to inform a subsequent pilot or implementation study.

### Research Questions

This study will address the following 6 research questions (RQs):

- RQ 1—what proportion of patients are willing to use head-mounted VR technology during routine dental care, and what factors influence willingness?
- RQ 2—what are the patients’ subjective experiences of using the head-mounted VR technology as measured through usability (System Usability Scale; SUS) and qualitative accounts?
- RQ 3—what are patients’ perceptions of the potential for head-mounted VR technology to enhance their experience during dental care?
- RQ 4—how willing are dental staff to trial and support VR technology use in their practice?
- RQ 5—what is the dental staff’s subjective experience of using the head-mounted VR technology during care provision?
- RQ 6—what are the dental staff’s perceptions of the potential for head-mounted VR technology to improve the patient experience during dental care?

## Methods

This feasibility study will use a mixed methods convergent design guided by the SPIRIT (Standard Protocol Items: Recommendations for Interventional Trials) 2013 checklist for interventional trials (Multimedia Appendix 1) [36].

### Ethical Considerations

This research was approved by the Peninsula Health Human Research Ethics Committee (project ID117565; Multimedia Appendix 2). All participants will be required to provide informed consent before enrollment. Participation is voluntary, and individuals can withdraw at any time without impact on their care or employment.

All data will be deidentified before being entered into the data analysis software at the end of the data collection period. No identifiable information will be published or reported.

Electronic data will be stored in a password-protected cloud storage system provided by Monash University. Hard-copy files will be kept in a locked filing cabinet. Access to the data will be restricted to researchers only. The storage of collected data will comply with Peninsula Health policies and Monash University regulations, and the data will be accessible only to study investigators. All data will be retained for at least 5 years following the study publication date. After this period, all electronic files will be permanently deleted, and any paper files will be shredded and placed in a secure document bin for disposal at Monash University.

Adverse events will be monitored, including VR sickness, dizziness, distress, or device fit discomfort. Stop rules include immediate headset removal upon any adverse reaction, with events logged and patients offered standard care without the device.

### Lived Experience Advisor Involvement

People with lived experience of disability and health conditions and community-based primary health care organizations in the health and disability sectors are actively involved in this project. This includes a formal collaboration with a lived experience advisor, who is an adult with cerebral palsy, and access to community advisory groups available through the collaborating health care network [37]. The project lived experience advisor will contribute to all components of the project, including the co-design of accessible recruitment materials, participant engagement strategies, and inclusive evaluation methods, providing specific paid deliverables, including co-design workshops and easy-read brief development. His lived experience of significant disability and assistive technology ensures that authentic disability perspectives guide all project phases from protocol development to dissemination. Lived experience involvement follows GRIPP2-SF (Guidance for Reporting Involvement of Patients and the Public 2–Short Form) reporting: the advocate will codesign recruitment materials and evaluation methods, with input directly shaping content selection and accessibility protocols through documented workshop outcomes.

### Settings

The study will be conducted at a community dental clinic, which is part of a public health network located at a community-based health and well-being hub in Victoria, Australia. This community-facing facility has been designed to deliver integrated health and well-being services. It hosts over a dozen organizations focused on intergenerational health, disability support, and inclusive health and well-being care. Within the hub, the community dental service provides general dental treatment to people of all ages in an accessible environment via prebooked appointments.

As part of this protocol development, 4 dental staff members from the hub were invited to an introductory session led by the study’s chief investigator. They were informed of the proposed study design and had the opportunity to trial Smileyscope. They also consulted on the preparation of 1 of the 2 dental chairs that will be used for the VR trials following the clinical sanitation guidelines of the dental service and also following the clinical sanitation guidelines of Smileyscope. It was explained at this time that the feasibility pilot of the VR-enabled treatment would be used during routine dental checkups but would be excluded from complex or emergency dental surgeries. Infection control protocols include disposable face shields or covers, antimicrobial wipe-down between uses, and designated downtime for sterilization compliance.

### Participants

Two distinct groups will be recruited.

- Group 1—adult patients aged ≥18 years who can provide informed consent for dental treatment and research participation. Exclusion criteria include emergency dental needs, contraindications for VR use, or inability to complete study instruments. The term “adult patients” is used operationally to reflect the real-world intake model of public dental services, in which adults may present with a diverse range of abilities or experience health conditions or disabilities. These may include physical, sensory, cognitive, psychosocial, and chronic health conditions or disabilities, which may be permanent, episodic, diagnosed, or self‑identified [38-40]. All eligible patients visiting the Peninsula Health dental services will be invited to participate in the study. Both patients coming in for their first visit or subsequent dental appointments will be included. Given the current volume of local patients, this research aims to recruit 50 participants for group 1 from October 2025 to March 2026.
- Group 2—dental staff, comprising dentists, dental nurses, and other staff working at participating Peninsula Health sites during the VR trial period. All Peninsula Health dental staff who have been present at a participating Peninsula Health site will be invited to participate in the study. Given the staffing allocation at dental sites, we aim to recruit up to 10 dental staff members for group 2.

### Recruitment

Recruitment flyers will be placed on trays in the waiting room of the dental service at both the Healthy Futures Hub and the adjacent Belvedere Community Centre. Dental reception staff will also invite patients to consider using VR during their dental care. The VR intervention will be offered across multiple weekdays between October 2025 and December 2025 using a schedule established in consultation with the local dental team to avoid congestion and ensure adequate time for patients and staff to engage with the technology. Patients can choose to trial the VR headset during their current visit or defer participation to a future appointment.

An experienced dental staff member was appointed by the Peninsula Health integrated and dental care manager to implement the VR trials in dental care as her rostered working day was the scheduled VR trial day (Fridays).

### Study Intervention

**Figure 1:**
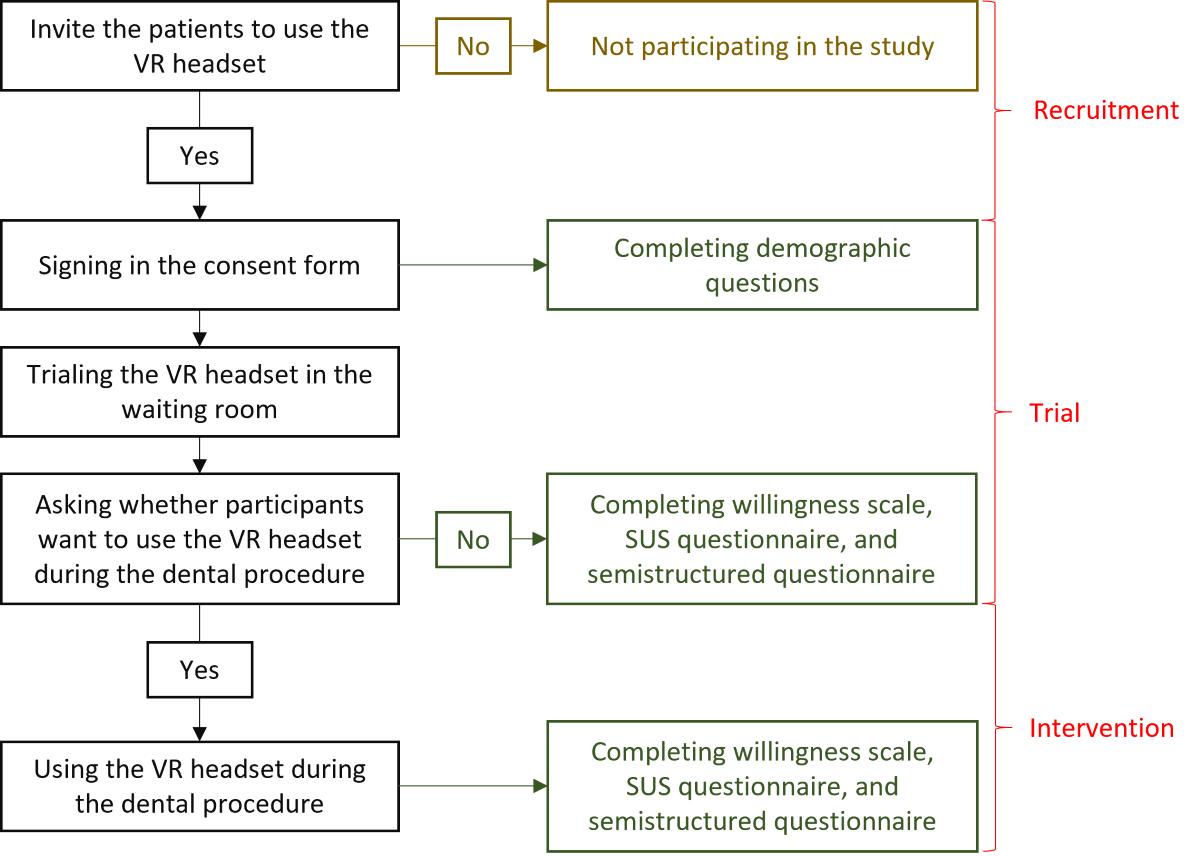
Patient recruitment and intervention. SUS: System Usability Scale; TAM: technology acceptance model; VR: virtual reality.

On designated VR trial days, a research team member will approach patients and provide further information about the study, including a verbal explanation and printed versions of the project explanatory statement, for them to consider consenting to participate in the study (Figure 1). They can then choose to trial the VR headset in the waiting room and during dental treatment.

**Figure 2:**
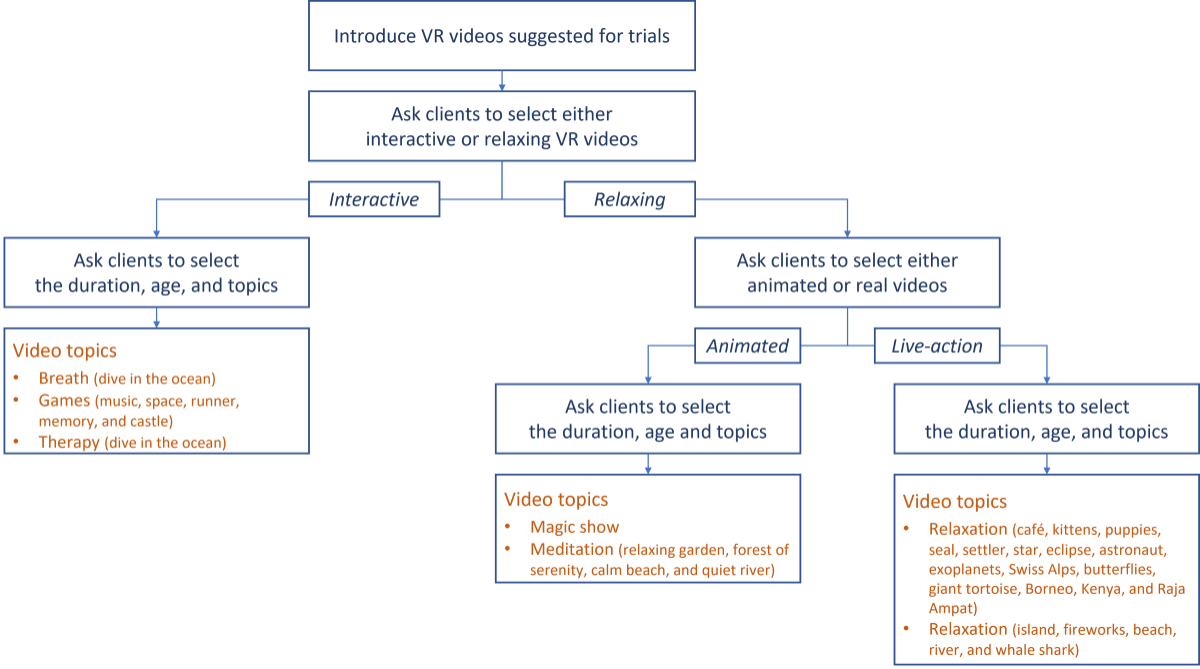
Map for selecting the experience on Smileyscope. VR: virtual reality.

To initiate the experience in the waiting room, a researcher will first clean the headset using an antimicrobial wipe and then power it on by holding the red button on the front display. Using the touch screen interface, the researcher will select a suitable program based on the patient’s age group, personal interest topic, and desired duration (rapid, short, medium, or long). Volume and language settings can also be adjusted, and for certain programs, the procedure site must be confirmed before proceeding (Figure 2).

Once the program is selected, the researcher will introduce the headset to the patient and ensure that the patient is in a safe and comfortable position. The headset will then be placed over the patient’s eyes, with straps adjusted for a snug but comfortable fit. Patients who wear glasses can typically keep them on. After connecting the faceplate, the researcher will triple-click the green play button to start the VR experience. A chime will confirm activation, and verbal cues will guide the patient through the session. Upon completion, the headset will be carefully removed, cleaned, and recharged for future use.

The patients will then be asked whether they would like to continue wearing the VR headset during their dental procedure. Those who decline will be invited to complete a visual analog scale (VAS) and semistructured interviews to capture their perspectives on VR use in dental care. Patients who agree to use the headset will proceed with the VR experience during their dental treatment and subsequently complete the VAS, the SUS, the technology acceptance model (TAM) questionnaire, and the semistructured questionnaire.

### Outcome Measurements

Primary outcomes include the feasibility, acceptability, and usability of the VR intervention during routine dental care from both the patient and dental staff perspectives.

The SUS is a widely used tool for assessing the usability of digital systems [41]. It consists of 10 items that measure users’ subjective perceptions of system usability, including aspects such as complexity, ease of use, and confidence in use. Each item is rated on a 5-point Likert scale ranging from 1 (“strongly disagree”) to 5 (“strongly agree”). The SUS provides a single score from 0 to 100, which reflects overall usability. It is particularly suitable for health care settings due to its brevity, reliability, and ease of administration, typically requiring less than 5 minutes to complete.

The TAM is a theoretical framework used to evaluate user acceptance of technology [42]. The adapted TAM questionnaire for this study includes 3 core constructs: perceived usefulness, attitude toward use, and behavioral intention to use. Each construct is measured using 3 to 4 items rated on a 5-point Likert scale. Perceived ease of use, another TAM construct, is assessed through overlapping items in the SUS. This combined approach enables the study to assess both the usability and acceptance of the Smileyscope VR headset among dental staff.

The SUS-only version (Multimedia Appendix 3) is intended for the patients as it focuses on usability, which is central to understanding how patients experience the Smileyscope VR headset. The combined SUS and TAM version (Multimedia Appendix 4) is designed for the staff as it assesses technology acceptance, including intent to use, perceived usefulness, and attitudes, which are more relevant for staff, who decide whether to adopt the technology in practice.

In addition, a separate 7-point VAS (Multimedia Appendix 5) will be developed to assess the willingness of both patients and dental staff to use VR headsets during dental treatments. This scale will range from −3 (indicating no willingness at all) to +3 (indicating a high level of willingness).

A brief demographic survey and semistructured interviews will also be used with both groups. Indicative questions to be used in the short interview are provided in Multimedia Appendix 6.

Disability or health condition status will be documented primarily via patient self-report in the demographic survey or in the interview [43]. Formal diagnostic verification is not required, consistent with disability-inclusive research principles and public health care practice, where functional experience and access needs are prioritized over diagnostic categorization.

Complementary performance indicators will also be collected, including the type and length of procedures and the duration of VR headset use for each participant.

### Data Collection

Table 1 summarizes the alignment between each RQ, the target participant group, the instruments used for data collection, and the timing of measurement throughout the study. This mapping ensures that feasibility, usability, willingness, and experiential outcomes related to VR use in dental care are captured systematically from both the patient and dental staff perspectives at appropriate stages of the trial.

**Table 1:** Data collection.

RQ^a^	Target participants	Measurement tool	Collection period
RQ 1: what proportion of patients are willing to use head-mounted VR^b^ technology during routine dental care, and what factors influence willingness?	Patients	VAS^c^	After the trial and after the intervention
RQ 2: what are the patients’ subjective experiences of using the head-mounted VR technology as measured through usability (SUS^d^) and qualitative accounts?	Patients	SUS	After the intervention
RQ 3: what are patients’ perceptions of the potential for head-mounted VR technology to enhance their experience during dental care?	Patients	Semistructured questionnaire	After the intervention
RQ 4: how willing are dental staff to trial and support VR technology use in their practice?	Dental staff	VAS	Before the trial
RQ 5: what is the dental staff’s subjective experience of using the head-mounted VR technology during care provision?	Dental staff	SUS and TAM^e^ questionnaire	After the intervention
RQ 6: what are the dental staff’s perceptions of the potential for head-mounted VR technology to improve the patient experience during dental care?	Dental staff	Semistructured questionnaire	After the intervention

^a^RQ: research question.

^b^VR: virtual reality.

^c^VAS: visual analog scale.

^d^SUS: System Usability Scale.

^e^TAM: technology acceptance model.

### Data Management

The demographic survey, VAS, SUS, and TAM questionnaire, and semistructured questionnaire will contain reidentifiable information, with a participant code applied to each data collection sheet.

Participants can complete the survey in hard copy and return it to the researchers in person, send it via mail, or scan and email it to the research group. Alternatively, the survey can be completed electronically and emailed to the research group.

### Statistical Considerations and Planned Analysis

The thematic analysis framework by Braun and Clarke [44] will be used to analyze the qualitative data gathered from the semistructured questionnaire. One researcher will enter the data gathered from the semistructured interviews according to the themes identified in each question. A second researcher will verify the accuracy of the data, whereas a third researcher will resolve any conflicts in data interpretation.

The thematic analysis will involve the following steps:

- Data familiarization—reading the responses multiple times to grasp the overall content
- Code generation—assigning labels or “codes” to key ideas or patterns in the data
- Review and refinement of themes—revisiting the data to ensure that the themes accurately represent the content and capture essential aspects of the feedback
- Presentation of results—description of the themes and how they relate to the RQs or objectives

Demographic data will be analyzed using descriptive statistics.

The SUS will be analyzed using the standard scoring method described by Brooke [45]. Each of the 10 items will be scored on a 5-point Likert scale. For positively worded items (odd numbered), the score contribution is the scale position minus 1; for negatively worded items (even numbered), the contribution is 5 minus the scale position. The total score is then multiplied by 2.5 to yield a usability score ranging from 0 to 100. Higher scores indicate better perceived usability. Descriptive statistics, including means and SDs, will be calculated to assess overall usability and variability in staff responses.

The TAM questionnaire will be analyzed by calculating the mean and SD for each of the 3 core constructs: perceived usefulness, attitude toward use, and behavioral intention to use. Perceived ease of use will be inferred from overlapping SUS items. A staff member will be considered to have accepted the technology if their mean score across behavioral intention to use items is of ≥4 (on a 5-point scale). The proportion of staff members meeting this threshold will be calculated. The TAM data will also be used to explore correlations between constructs and identify factors influencing acceptance.

Primary feasibility indicators include recruitment rate (≥60% consent), completion rate (≥80% SUS completion), and usability threshold (mean SUS ≥68). The primary analysis will be descriptive, with 95% CIs reported.

Given the feasibility nature of the study and the anticipated sample size, formal stratification by disability subtype is not planned [38,46]. However, to support interpretability and transparency, participants’ self‑reported disability or health condition characteristics will be captured descriptively. Descriptive categories may include physical, sensory, cognitive, psychosocial, and chronic health conditions, recognizing that participants may identify across multiple domains. These data will be reported using descriptive summaries and narrative interpretation rather than inferential subgroup analyses [40,46]. Where sample size permits, exploratory noncomparative observations may be presented to inform the design of future pilot or implementation studies.

### Expected Outcomes

This study will generate foundational evidence in Australia on the feasibility, usability, and acceptability of integrating VR technology into adult public dental care offered within a community dental service. By evaluating the Smileyscope VR headset, the research will provide critical insights into patient and clinician experiences, willingness to use the technology, and its integration into routine dental workflows.

Key outcomes will include the development of an implementation toolkit containing costed solutions to common VR integration barriers, such as staff training protocols and accessibility modifications. Additionally, the study will produce evidence-informed recommendations for scaling sustainable VR integration in public dental services supported by specific cost-benefit analyses. These findings will directly inform service design improvements and contribute to the growing evidence base on digital health innovation in dentistry.

## Results

The study received institutional funding support in 2025 and Peninsula Health Human Research Ethics Committee approval before participant enrollment (project ID117565). Data collection is planned for October 2025 to March 2026 across participating public dental clinics. Initial feasibility and usability results are targeted for submission in mid‑2026. Recruitment data have been cleaned and analyzed at this time.

## Discussion

### Expected Findings

This paper outlines an a priori research protocol for a feasibility study designed to evaluate the integration of head-mounted VR technology into routine public dental care for adults with disabilities or health conditions. The intervention will use the Smileyscope VR headset to deliver immersive, calming experiences aimed at reducing anxiety and enhancing patient engagement. By assessing usability, acceptability, and willingness to use VR from both the patient and clinician perspectives, the study aims to generate foundational evidence to inform future implementation and scale-up efforts.

Previous research has demonstrated the effectiveness of VR in pediatric dental and hospital settings, particularly in reducing procedural anxiety and improving patient cooperation [14-17,28-32]. However, there is limited evidence on the use of VR in adult dental care, especially among populations with disabilities or chronic health conditions. This study addresses that gap by focusing on adult patients in community-based public dental services using a co-designed approach that incorporates lived experience and inclusive evaluation methods.

The protocol also builds on broader health care literature that supports VR as a tool for distraction, pain management, and procedural efficiency [20-22]. Unlike many existing studies that focus on single-session outcomes or pediatric populations, this study is designed to explore the feasibility of integrating VR into routine workflows and understand the perspectives of both patients and clinicians.

### Benefits and Risks

The anticipated benefits of VR integration include reduced dental anxiety, improved patient cooperation, and enhanced clinician focus during procedures. These outcomes may contribute to more efficient care delivery and improved patient satisfaction. For dental staff, the use of VR may foster a more supportive work environment by reducing procedural stress and improving patient engagement.

Potential risks include discomfort or disorientation for some patients, particularly those with vestibular or other sensory sensitivities or cognitive communication changes. The headset may not be suitable for all procedures, and its use requires appropriate screening and staff training. Technical issues, hygiene protocols, and workflow disruptions are also considerations that will be addressed through the implementation toolkit.

### Limitations of the Study and Future Directions

As a feasibility study, this protocol is limited by its short duration (October 2025 to December 2025) and relatively small sample size. The intervention is restricted to routine dental checkups and excludes emergency care and complex procedures. Only patients capable of providing informed consent will be included, which may limit generalizability to all populations with cognitive communication changes.

Future research should explore the long-term impact of VR integration on oral health outcomes, treatment adherence, and clinician performance. Studies involving broader and more diverse populations, including children, older adults, and individuals with cognitive communication impairments, are needed to assess the scalability and inclusivity of VR interventions. Additionally, further investigation into cost-effectiveness and integration into other areas of primary and allied health care will be essential for sustainable adoption.

### Conclusions

This protocol presents a feasibility study that will evaluate the use of head-mounted VR technology to reduce dental anxiety and improve care experiences for adults with disabilities. By assessing usability, acceptability, and willingness to use Smileyscope in public dental settings, the study will generate foundational evidence to inform scalable, inclusive models for VR integration into oral health care. The findings will support service design improvements and contribute to the digital health innovation literature in dentistry.

## Appendix

Multimedia Appendix 1 SPIRIT checklist.

Multimedia Appendix 2 Ethics approval letter.

Multimedia Appendix 3 System Usability Scale for patients.

Multimedia Appendix 4 System Usability Scale and technology acceptance model questionnaire for staff.

Multimedia Appendix 5 Visual analog scale for willingness.

Multimedia Appendix 6 Semistructured questions.

## Abbreviations

GRIPP2-SF: Guidance for Reporting Involvement of Patients and the Public 2–Short Form
RQ: research question
SPIRIT: Standard Protocol Items: Recommendations for Interventional Trials
SUS: System Usability Scale
TAM: technology acceptance model
VAS: visual analog scale
VR: virtual reality
