# Supplementary material for: Anxiety-Free Public Dentistry for Adults With Disabilities by Using Head-Mounted Virtual Reality Technology: Protocol for a Feasibility Mixed Methods Study
Source: JMIR Res Protoc. 2026 Feb 13;15:e85916. doi: 10.2196/85916 (PMC12949397; doi:10.2196/85916)
Supplement: Multimedia Appendix 3 [file resprot_v15i1e85916_app3.docx]

**System Usability Scale (SUS) - for Clients**

You are invited to participate in a research project conducted by Monash University in collaboration with Peninsula Health. The purpose of this research is to explore the perspectives of clinicians and adult patients on the acceptability, feasibility and usability of the SmileyScope VR device during routine dental care.

This survey is being given/sent to clients aged over 18 years who can provide consent for dental treatment.

It should take approximately 5 minutes to complete.

By completing the survey, you will provide information on your perspectives on the feasibility of the SmileyScope VR that may assist in understanding and supporting VR applications in dental treatment.

Your name is not recorded anywhere on the survey.

If you wish to participate in the research project, please complete the survey. By completing the survey, you are telling us that you

- Understand what you have read
- Consent to take part in the research project
- Consent to the use of your survey data as described

Participation in this survey is voluntary; if you do not wish to take part, you do not have to.

Once you have submitted your responses, they remain in the data collected by the survey. If you decide that you would like to withdraw from participation after you have submitted your answers, your data cannot be removed, as it was not identified.

Results of this survey will be available on request from Dr Trung Dung Bui ([TrungDung.Bui@monash.edu](mailto:TrungDung.Bui@monash.edu)), Monash University and will be submitted for publication and presented at conferences and seminars.

The ethical aspects of this project have been approved by the Peninsula Health Human Research Ethics Committee.

If you have any questions regarding this survey, you may contact the researcher, Dr Trung Dung Bui ([TrungDung.Bui@monash.edu](mailto:TrungDung.Bui@monash.edu)).

If you have any complaints about any aspect of this research project, the way it is being conducted or any questions about being a research participant in general, you may contact:

Manager, Office for Research, Peninsula Health

Telephone**:** 9784 2679

Email: researchethics@phcn.vic.gov.au

Please rate your agreement with the following statements based on your experience using the Smileyscope VR headset during your dental visit.

|  |  | Strongly Disagree |  |  |  | Strongly Agree |
| --- | --- | --- | --- | --- | --- | --- |
|  | I would like to use Smileyscope VR again during dental visits. |  |  |  |  |  |
|  |  | 1 | 2 | 3 | 4 | 5 |
|  | I found Smileyscope VR unnecessarily complex. |  |  |  |  |  |
|  |  | 1 | 2 | 3 | 4 | 5 |
|  | I thought Smileyscope VR was easy to use. |  |  |  |  |  |
|  |  | 1 | 2 | 3 | 4 | 5 |
|  | I needed help from staff to use Smileyscope VR. |  |  |  |  |  |
|  |  | 1 | 2 | 3 | 4 | 5 |
|  | I felt Smileyscope VR worked well with the dental care I received. |  |  |  |  |  |
|  |  | 1 | 2 | 3 | 4 | 5 |
|  | I thought Smileyscope VR was inconsistent in how it worked. |  |  |  |  |  |
|  |  | 1 | 2 | 3 | 4 | 5 |
|  | I think most people would learn to use Smileyscope VR quickly. |  |  |  |  |  |
|  |  | 1 | 2 | 3 | 4 | 5 |
|  | I found Smileyscope VR cumbersome to use. |  |  |  |  |  |
|  |  | 1 | 2 | 3 | 4 | 5 |
|  | I felt confident using Smileyscope VR. |  |  |  |  |  |
|  |  | 1 | 2 | 3 | 4 | 5 |
|  | I needed to learn a lot before I could use Smileyscope VR. |  |  |  |  |  |
|  |  | 1 | 2 | 3 | 4 | 5 |

**For researchers only**

The name of the dental procedure the client received: ___________________________

____________________________________________________________________________________

The duration of the procedure: _____________ minutes.

The duration of the Smileyscope VR usage: _____________ minutes.
