# Supplementary figures and images for: Anxiety-Free Public Dentistry for Adults With Disabilities by Using Head-Mounted Virtual Reality Technology: Protocol for a Feasibility Mixed Methods Study
Source: JMIR Res Protoc. 2026 Feb 13;15:e85916. doi: 10.2196/85916 (PMC12949397; doi:10.2196/85916)

**Willingness Scale**


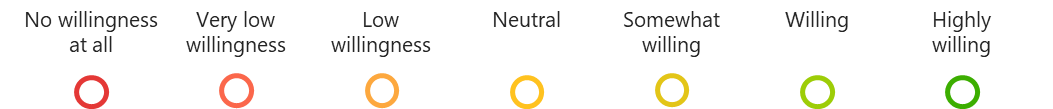

Supplement: Multimedia Appendix 5 [file resprot_v15i1e85916_app5.docx]
