# Supplementary material for: Anxiety-Free Public Dentistry for Adults With Disabilities by Using Head-Mounted Virtual Reality Technology: Protocol for a Feasibility Mixed Methods Study
Source: JMIR Res Protoc. 2026 Feb 13;15:e85916. doi: 10.2196/85916 (PMC12949397; doi:10.2196/85916)
Supplement: Multimedia Appendix 6 [file resprot_v15i1e85916_app6.docx]

**Semi-structured questions for Patient and Dental staff interviews**

1. How familiar are you with virtual reality (VR) technology, and have you ever used it before? If so, in what context?
2. What are your initial thoughts on the idea of using VR technology during dental procedures?
3. Do you have any concerns or reservations about the use of VR technology in dental procedures? If so, what are they?
4. In what ways do you think VR technology could improve experiences during dental care?
5. How do you think VR technology could be integrated into dental care to make the experience more comfortable or enjoyable for patients?
6. Would you be willing to try the use of VR technology during a dental procedure? Why or why not?
