# Supplementary material for: Anxiety-Free Public Dentistry for Adults With Disabilities by Using Head-Mounted Virtual Reality Technology: Protocol for a Feasibility Mixed Methods Study
Source: JMIR Res Protoc. 2026 Feb 13;15:e85916. doi: 10.2196/85916 (PMC12949397; doi:10.2196/85916)
Supplement: Multimedia Appendix 2 [file resprot_v15i1e85916_app2.pdf]

Peninsula Health Human Research Ethics Committee

ETHICS APPROVAL FOR AMENDMENT

Dr Libby Callaway
Level 4, Building G,
Monash University
Peninsula Campus
Frankston
3199

18 September 2025

Dear Dr Libby Callaway,

**Project Title**	Virtual Reality Application for Adult Dental Treatment: A Feasibility Study
**Project ID**	117565
**Review Reference**	AM/117565/PH-2025-498561(v1)

I am pleased to advise that the amendment to the above project has received ethical approval from Peninsula Health Human Research Ethics Committee (HREC).

This HREC is organised and operates in accordance with the National Health and Medical Research Council's (NHMRC) *National Statement on Ethical Conduct in Human Research* (2023), and all subsequent updates, and in accordance with the *Note for Guidance on Good Clinical Practice (CPMP/ICH/135/95)*, the Health Privacy Principles described in the *Health Records Act 2001* (Vic) and Section 95A of the *Privacy Act 1988* (and subsequent Guidelines).

This amendment approval applies for all sites for which Peninsula Health HREC has issued ethical approval.

This amendment approval is subject to compliance with the conditions imposed by the reviewing HREC at the time of (a) the original ethics approval and (b) any amendment approvals prior to this.

Approved Documents

The following documents have been reviewed and approved:

Document Type	File Name	Date	Version
Protocol (Tracked)	VR in dental treatment protocol - Pen Health - V2.0 20250829 - highlighted	01/09/2025	2.0
	VR in dental treatment		

Document Type	File Name	Date	Version
Protocol	protocol - Pen Health - V2.0 20250829 - clean	01/09/2025	2.0
PARTICIPANT INFORMATION AND CONSENT FORM (TRACKED)	3.1 PICF-interventional-for-self - Clients 20250829 - highlighted	01/09/2025	2.0
PARTICIPANT INFORMATION AND CONSENT FORM (TRACKED)	3.2 PICF-interventional-for-self - Staff 20250829 - highlighted	01/09/2025	2.0
Participant information and consent form	3.1 PICF-interventional-for-self - Clients 20250829 - clean	01/09/2025	2.0
Participant information and consent form	3.2 PICF-interventional-for-self - Staff 20250829 - clean	01/09/2025	2.0
Other	4.5.1 SUS-TAM for Staff 20250828	01/09/2025	1.0
Other	4.5.2 SUS for Clients 20250828	01/09/2025	1.0
Curriculum vitae	6.5 CV- Jane Blandy	01/09/2025	1.0

Research Governance Amendment

You are required to provide a copy of this amendment approval letter to the principal investigator of each site that is affected by the amendment and is covered by the Peninsula Health HREC approval. The principal investigator should provide a copy to their site research governance officer (RGO). An amendment to the site research governance/site specific assessment (SSA) authorisation may be required.

If any matters arise regarding conduct of the research at this site, please contact the RGO via email at rgo@phcn.vic.gov.au or by phone on 9784 2680.

Yours sincerely,

Office for Research
Peninsula Health
